# Hyperlactatemia is a predictor of mortality in patients undergoing continuous renal replacement therapy for acute kidney injury

**DOI:** 10.1186/s12882-023-03063-y

**Published:** 2023-01-14

**Authors:** Seong Geun Kim, Jinwoo Lee, Donghwan Yun, Min Woo Kang, Yong Chul Kim, Dong Ki Kim, Kook-Hwan Oh, Kwon Wook Joo, Yon Su Kim, Seung Seok Han

**Affiliations:** Department of Internal Medicine, Seoul National University College of Medicine, 103 Daehak-ro, Jongno-gu, 03080 Seoul, Korea

**Keywords:** Acute kidney injury, Continuous renal replacement therapy, Mortality, Lactate

## Abstract

**Background:**

Hyperlactatemia occurs frequently in critically ill patients, and this pathologic condition leads to worse outcomes in several disease subsets. Herein, we addressed whether hyperlactatemia is associated with the risk of mortality in patients undergoing continuous renal replacement therapy (CRRT) due to acute kidney injury.

**Methods:**

A total of 1,661 patients who underwent CRRT for severe acute kidney injury were retrospectively reviewed between 2010 and 2020. The patients were categorized according to their serum lactate levels, such as high (≥ 7.6 mmol/l), moderate (2.1–7.5 mmol/l) and low (≤ 2 mmol/l), at the time of CRRT initiation. The hazard ratios (HRs) for the risk of in-hospital mortality were calculated with adjustment of multiple variables. The increase in the area under the receiver operating characteristic curve (AUROC) for the mortality risk was evaluated after adding serum lactate levels to the Sequential Organ Failure Assessment (SOFA) and the Acute Physiology and Chronic Health Evaluation (APACHE) II score-based models.

**Results:**

A total of 802 (48.3%) and 542 (32.6%) patients had moderate and high lactate levels, respectively. The moderate and high lactate groups had a higher risk of mortality than the low lactate group, with HRs of 1.64 (1.22–2.20) and 4.18 (2.99–5.85), respectively. The lactate-enhanced models had higher AUROCs than the models without lactates (0.764 vs. 0.702 for SOFA score; 0.737 vs. 0.678 for APACHE II score).

**Conclusions:**

Hyperlactatemia is associated with mortality outcomes in patients undergoing CRRT for acute kidney injury. Serum lactate levels may need to be monitored in this patient subset.

## Introduction

Hyperlactatemia is attributable to either excessive production of lactate due to hypoxia or impaired clearance [1–3]; the condition is strongly associated with worse outcomes in critically ill patients [4–6]. Previous studies have suggested that serum lactate is a useful biomarker in risk stratification, particularly for septic patients [7–9]. Therefore, the Surviving Sepsis Campaign guidelines continue to recommend the measurement of lactates in patients with documented or suspected sepsis to help identify patients at high risk of death and to confer suitability for intensive care [10]. Lactate levels have been evaluated as a prognostic factor in other pathologic conditions such as trauma, postoperation, and burns, wherein hyperlactatemia was associated with high mortality rates [11–14].

Continuous renal replacement therapy (CRRT) is often the first-choice treatment for acute kidney injury (AKI) patients requiring RRT, especially when hemodynamics are unstable [15]. Despite advances in CRRT techniques, the overall mortality rates have not decreased by more than 50% over the past two decades [16, 17]. Accordingly, precisely assessing and monitoring the patient’s status is essential to achieving improved outcomes. Several patient parameters, such as age, comorbidities, fluid status, and certain indices, such as sequential organ failure assessment (SOFA), Acute Physiology and Chronic Health Evaluation (APACHE) II, and the abbreviated Mortality Scoring system for AKI with CRRT (MOSAIC) have been shown to correlate with the outcomes of patients who undergo CRRT [17–19]. Some previous studies demonstrated the trend of acidosis in early period of CRRT was associated with mortality [20, 21].

However, studies on the application of lactates to predict outcomes in patients receiving CRRT are lacking, and thus, it is unknown whether monitoring lactates is needed after starting CRRT. Herein, we addressed this issue in a cohort of patients receiving CRRT due to AKI and added lactate levels into the severity index-based models to identify its synergistic predictability.

## Method

### Patient and data collection

The study was retrospective study of a cohort of 2,832 patients who underwent CRRT due to AKI at Seoul National University Hospital from June 2010 to December 2020. Among them, patients who were under 18 years old (*n* = 24) and were previously on dialysis because of end-stage kidney disease (*n* = 377) were excluded. Serum lactate levels at the time of CRRT initiation were unavailable in 770 patients, and thus, a total of 1,661 patients were ultimately analyzed.

Baseline information at the time of CRRT initiation was collected, such as age, sex, weight, cause of AKI (e.g., septic and nonseptic), initial CRRT settings (e.g., target dose, blood flow rate, and target ultrafiltration), mechanical ventilation, use of vasopressors, the division of intensive care unit, and severity indices (e.g., SOFA, APACHE II, and Charlson comorbidity index [CCI]). The SOFA, APACHE II, and CCI scores were calculated according to the original formulae [22–24]. We applied the mode of continuous venovenous hemodiafiltration to all cases. The patients were categorized into 3 groups according to the lactate levels, as follows: ≤ 2 mmol/l, the low group; 2.1–7.5 mmol/l, the moderate group; and ≥ 7.6 mmol/l, the high group. A cutoff value of 2 mmol/l was determined with a previous cohort result [25], and the moderate and high groups were divided by the median among the patients with > 2 mmol/l. The lactate clearance within 24 h was additionally obtained (*n* = 1,115) because this parameter is known to correlate with the mortality risk [26, 27]. The clearance was calculated using the following formula: 100 × (initial lactate level – 24-h lactate level) / initial lactate level.

### Outcomes

The primary outcome was all-cause mortality after CRRT until discharge. Additionally, mortality on CRRT and at discharge from the intensive care unit were evaluated. The CRRT mortality was defined when the patients were expired before the weaning from CRRT.

### Statistical analysis

Categorical and continuous variables are expressed as proportions and means ± standard deviations when they were normally distributed and as medians with interquartile ranges when they were not normally distributed, respectively. The normality of the distribution was analyzed using the Kolmogorov–Smirnov test. The chi-square test or Fisher’s exact test was used to compare categorical variables. Student’s t test or the Mann–Whitney U test was used for continuous variables with or without a normal distribution.

Restricted cubic spline model was applied to fit the relationship between serum lactate and mortality risks. Kaplan–Meier survival curves were drawn and compared between groups using a log-rank test. The hazard ratios (HRs) and confidence intervals for mortality outcomes were calculated using the Cox proportional hazard regression model. The odds ratios (ORs) and confidence intervals for mortality outcomes were calculated using logistic regression analysis in which multiple variables were adjusted. The adjusted variables were selected based on their significant association with mortality risk. The area under the receiver operating characteristic curve (AUROC) and DeLong test were used to evaluate the difference in discriminative power when the lactate value was added to the SOFA and APACHE II score-based models. All statistical analyses were performed using SPSS software (version 27; IBM, Armonk, NY, USA) and R software (version 3.5.1; R core team, Vienna, Austria). A *P*-value less than 0.05 was considered statistically significant.

## Results

### Patient characteristics

The mean patient age was 64 ± 15 years, and 37.8% of the patients were female. The proportion of patients with septic AKI was 58.6%. When the patients were divided into 3 groups by initial lactate levels, the proportion of septic AKI, and SOFA and APACHE II scores were significantly higher in the moderate and high lactate groups than in the low lactate group. Other baseline characteristics are presented in Table 1.

**Table 1:** Baseline characteristics of the patients

		Lactate groups			
Variables	All (*n* = 1,661)	Low (*n* = 317)	Moderate (*n* = 802)	High (*n* = 542)	*P*
Age (years)	63.6 ± 15.1	64.1 ± 15.8	64.2 ± 15.2	62.7 ± 14.7	0.144
Female (%)	37.8	36.0	35.0	41.4	0.041
Weight (kg)	65.3 ± 13.0	62.3 ± 13.7	62.2 ± 12.6	62.4 ± 13.1	0.542
Septic acute kidney injury (%)	58.6	54.6	55.8	63.3^†^	0.005
Target dose (ml/kg/hr)	41.0 ± 12.8	39.8 ± 12.9	39.8 ± 12.4	42.8 ± 13.4^†^	< 0.001
Blood flow rate (ml/min)	113.4 ± 24.8	114.1 ± 25.3	112.7 ± 24.0	113.6 ± 25.3	0.674
Target ultrafiltration (ml/d)	0 (–500–0)	0 (–500–0)	0 (–500–0)	0 (–500–0)	0.761
Mechanical ventilation (%)	77.2	62.8	76.2^‡^	84.9^‡^	< 0.001
Use of ≥ 3 vasopressors (%)	17.5	6.6	13.9^†^	26.0^‡^	< 0.001
ICU type (%)					0.110
Medical ICU	60.9	58.7	61.1	61.7	
Surgical ICU	18.8	21.8	17.7	18.6	
Cardiopulmonary ICU	1.5	2.2	2.1	0.6	
Emergency ICU	18.5	16.7	18.9	19.1	
COVID-19 ICU	0.2	0.6	0.3	0.0	
SOFA score	12.0 ± 3.7	9.9 ± 3.4	11.8 ± 3.6^‡^	13.1 ± 3.5^‡^	< 0.001
APACHE II score	26.5 ± 7.8	23.1 ± 7.3	26.1 ± 7.7^‡^	28.6 ± 7.6^‡^	< 0.001
CCI score	2 (1–3)	2 (1–3)	2 (1–3)	2 (1–3)	0.868

*ICU* intensive care unit, *COVID-19* COrona VIrus Disease-19, *SOFA* Sequential Organ Failure Assessment, *APACHE* Acute Physiology and Chronic Health Evaluation, *CCI* Charlson comorbidity index

^*^P < 0.05; ^†^*P* < 0.01; ^‡^*P* < 0.001 compared with the low lactate group

### Relationship between serum lactate and mortality

**Fig. 1:**
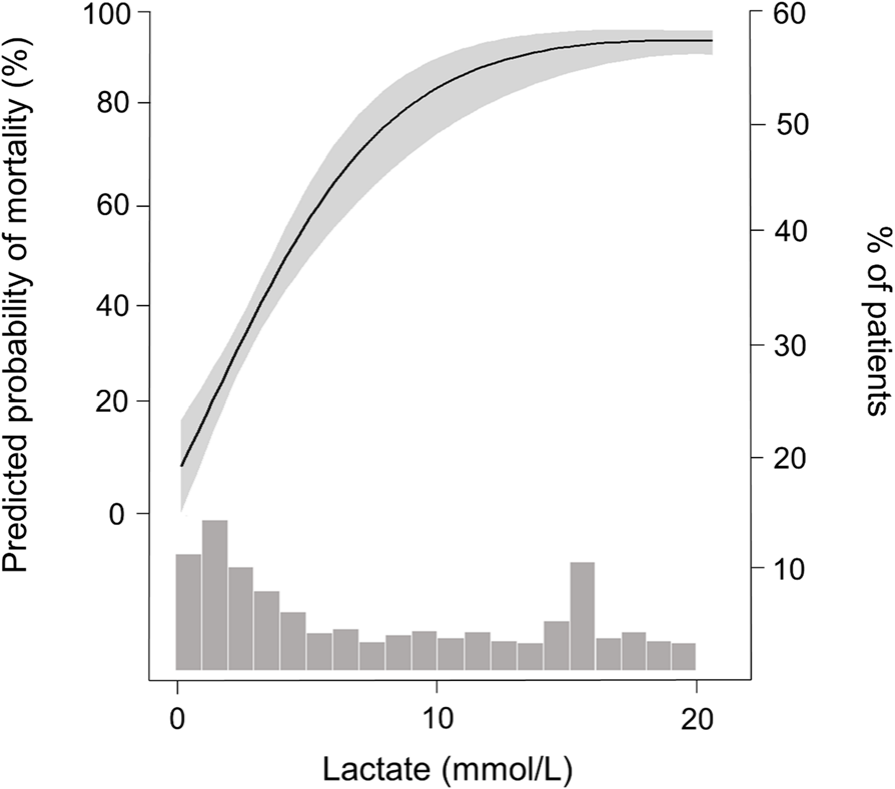
Nonlinear relationship between the initial lactate levels and the predicted risk of in-hospital mortality. The gray area indicates the 95% confidence interval. A histogram of cases is indicated on the right y-axis

**Fig. 2:**
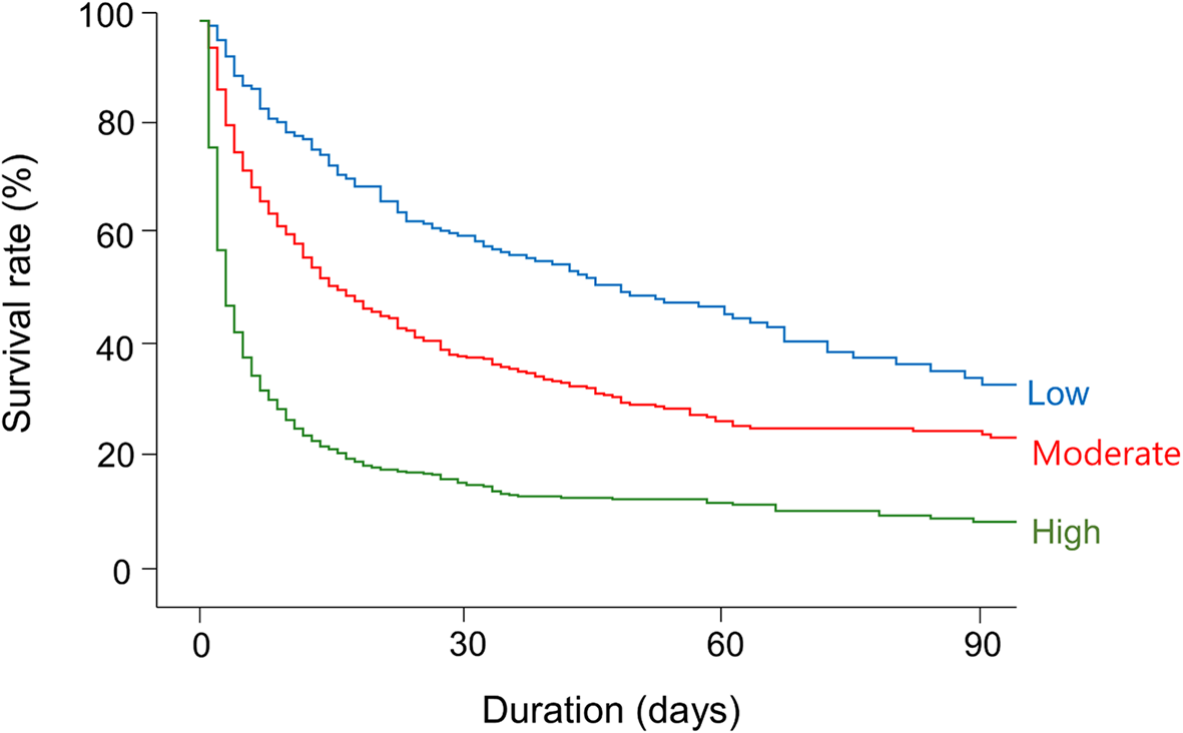
Kaplan–Meier survival curves according to the initial lactate levels

During a median follow-up period of 10 days (interquartile range, 3–28 days), 1,167 patients (70.3%) died. The mortality incidence was 26.7 deaths per 1,000 person-days. When univariable Cox regression model was applied, several variables, such as age, septic AKI, mechanical ventilation, use of ≥ 3 vasopressors, and scores of SOFA, APACHE II, and CCI were related to all-cause mortality in the present cohort (Table 2), and these were used in subsequent multivariable regression models for the adjustment. The restricted cubic spline curve displayed a positive nonlinear relationship between serum lactate and the mortality risks (Fig. 1). The mortality risks increased with the initial lactate level and reached a plateau in the high lactate group. Figure 2 shows Kaplan–Meier survival curves of the 3 lactate groups, and the survival rates among the groups were different (*P* < 0.001) (Fig. 2). Table 3 shows the ORs of mortality outcomes according to lactate levels. The risk of mortality outcomes increased depending on the increase in lactate levels independent of the effects of multiple variables.

**Table 2:** Factors related to all-cause mortality

Variables	Hazard ratio (95% CI)	*P*
Age (per 1 year)	1.006 (0.999–1.012)	0.099
Female (vs. male)	0.970 (0.861–1.093)	0.970
Body weight (per 1 kg)	0.998 (0.994–1.003)	0.431
Septic AKI (vs. non-septic AKI)	1.337 (1.187–1.506)	< 0.001
Target dose (per 10 ml/kg/hr)	1.020 (0.977–1.065)	0.368
Blood flow rate (per 10 ml/min)	1.013 (0.990–1.037)	0.278
Target ultrafiltration (per 1 L/day)	1.008 (0.932–1.090)	0.884
Mechanical ventilation (vs. none)	1.493 (1.288–1.730)	< 0.001
≥ 3 vasopressors (vs. < 3)	1.593 (1.382–1.837)	< 0.001
SOFA score (per 1 score)	1.132 (1.113–1.151)	< 0.001
APACHE II score (per 1 score)	1.055 (1.047–1.063)	< 0.001
CCI score (per 1 score)	1.014 (1.013–1.066)	< 0.001

*CI* confidence interval, *AKI* acute kidney injury, *SOFA* Sequential Organ Failure Assessment, *APACHE* Acute Physiology and Chronic Health Evaluation, *CCI* Charlson Comorbidity Index

**Table 3:** Risk of mortality according to lactate levels

Outcomes	Lactate levels	Unadjusted OR (95% CI)	*P*	Adjusted OR (95% CI)^a^	*P*
In-hospital mortality	Low	1 (Reference)		1 (Reference)	
	Moderate	2.29 (1.744–3.009)	< 0.001	1.64 (1.222–2.207)	0.001
	High	6.99 (5.137–9.515)	< 0.001	4.18 (2.985–5.847)	< 0.001
ICU mortality	Low	1 (Reference)		1 (Reference)	
	Moderate	2.25 (1.707–2.961)	< 0.001	1.55 (1.154–2.093)	0.004
	High	7.38 (5.474–9.942)	< 0.001	4.22 (3.048–5.830)	< 0.001
CRRT mortality	Low	1 (Reference)		1 (Reference)	
	Moderate	2.11 (1.596–2.798)	< 0.001	1.48 (1.099–2.004)	0.010
	High	7.45 (5.567–10.102)	< 0.001	4.45 (3.231–6.135)	< 0.001

Low, ≤ 2 mmol/l; moderate, 2.1–7.5 mmol/l; high, ≥ 7.6 mmol/l

*OR* odds ratio, *CI* confidence interval, *ICU* intensive care unit, *CRRT* continuous renal replacement therapy

^a^Adjusted for age, sex, body weight, septic acute kidney injury, mechanical ventilation, vasopressors, and scores of SOFA, APACHE II, and CCI

### Additive predictability of lactate in the scoring models

**Fig. 3:**
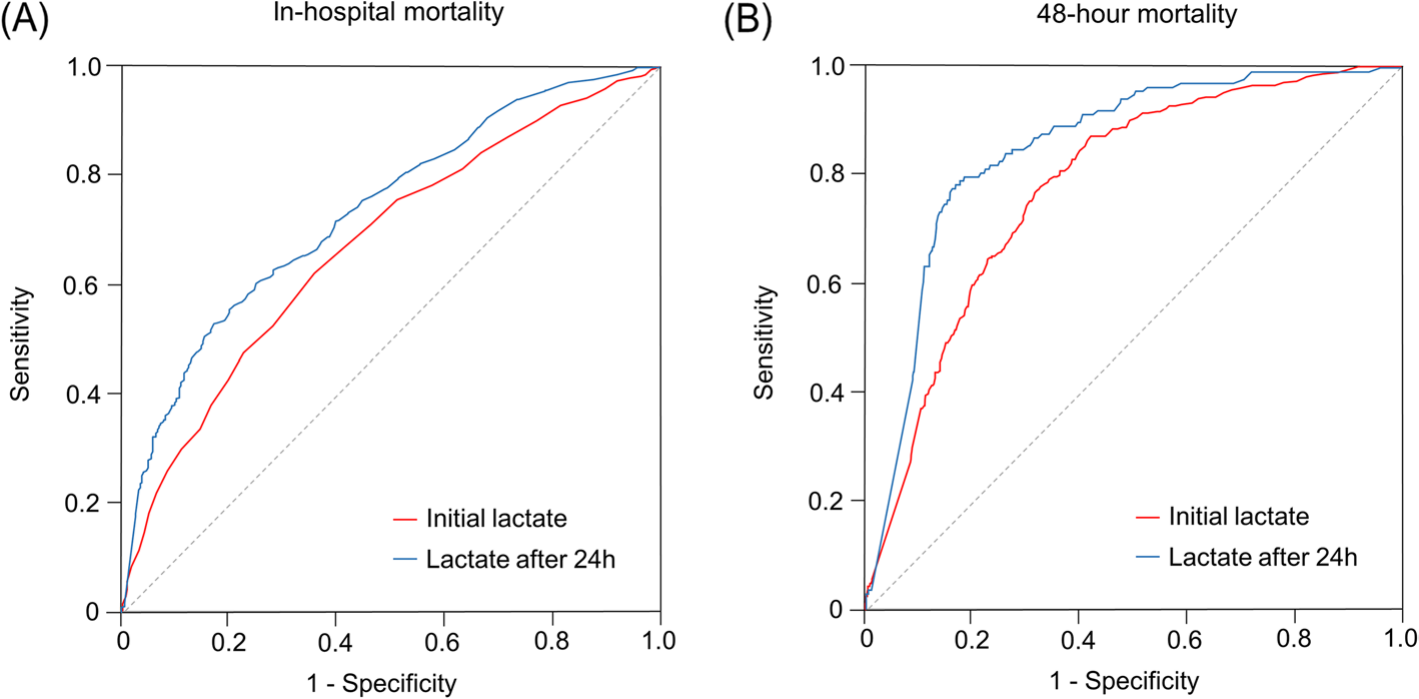
Receiver operating characteristic curves of serum lactates before and after 24 h of CRRT for (**A**) in-hospital and (**B**) 48-h mortality

**Fig. 4:**
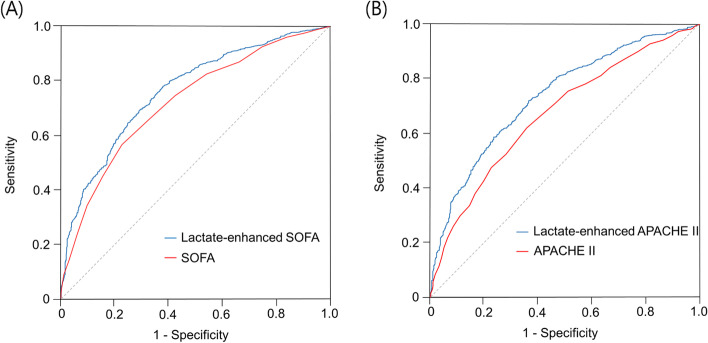
Comparison of the receiver operating characteristic curves for in-hospital mortality. **A** Between the Sequential Organ Failure Assessment (SOFA) model with and without lactate levels. **B** Between the Acute Physiology Assessment and Chronic Health Evaluation (APACHE) II models with and without lactate levels

Figure 3A shows the ROC curves for in-hospital mortality of serum lactates. The AUROCs in the model with initial lactates and the lactates after 24 h were 0.708 (0.681–0.734) and 0.734 (0.704–0.765), respectively. When 48-h mortality was set as the outcome, the AUROCs increased for initial lactates (0.779 [0.755–0.804]) and lactates after 24 h of CRRT (0.852 [0.822–0.883]) (Fig. 3B). We addressed the addition of initial lactates for predictability to the original SOFA and APACHE II scores for in-hospital mortality. The AUROC in the lactate-enhanced SOFA model was higher than that in the lactate-absent SOFA model (0.764 vs. 0.702, *P* < 0.001) (Fig. 4A). The lactate-enhanced APACHE II model had a greater AUROC than the model without lactate (0.737 vs. 0.678, *P* < 0.001) (Fig. 4B).

### Relationship between lactate clearance and mortality

**Fig. 5:**
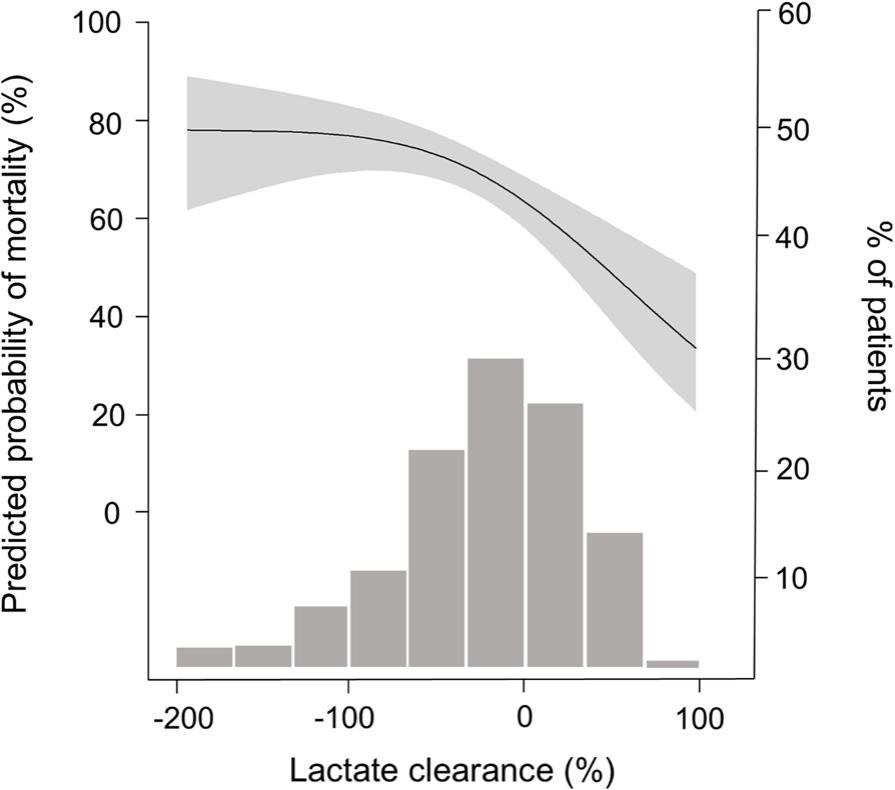
Nonlinear relationship between the lactate clearance within 24 h and the predicted risk of in-hospital mortality. The gray area indicates the 95% confidence interval. A histogram of cases is indicated on the right y-axis

**Fig. 6:**
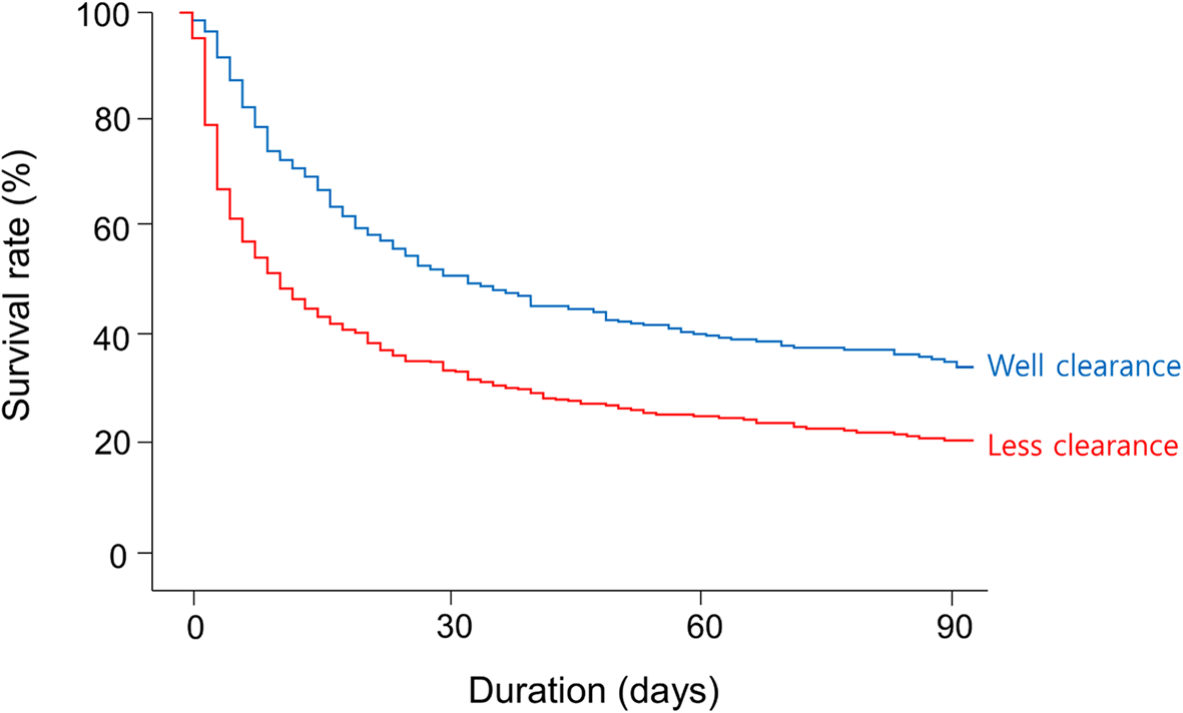
Kaplan–Meier survival curves according to lactate clearance within 24 h

The median value of lactate clearance was 0% (interquartile range, –33% to + 33%). When a restricted cubic spline model was applied, the risk of mortality correlated inversely with the lactate clearance value (Fig. 5). The mortality risks reached a plateau in the point where the initial lactate doubled. Patients were categorized into two groups: the well (clearance > 0%) and less (clearance ≤ 0%) clearance groups. Figure 6 shows the Kaplan–Meier survival curves of the two groups, and their survival rates were different (*P* < 0.001). The well clearance group was associated with lower mortality rates than the less clearance group despite the adjustment for multiple variables (Table 4).

**Table 4:** Risk of mortality according to lactate clearance

Outcomes	Lactate clearance	Unadjusted OR (95% CI)	*P*	Adjusted OR (95% CI)^a^	*P*
In-hospital mortality	Less clearance	1 (Reference)		1 (Reference)	
	Well clearance	0.51 (0.396–0.664)	< 0.001	0.51 (0.382–0.668)	< 0.001
ICU mortality	Less clearance	1 (Reference)		1 (Reference)	
	Well clearance	0.44 (0.343–0.560)	< 0.001	0.42 (0.317–0.544)	< 0.001
CRRT mortality	Less clearance	1 (Reference)		1 (Reference)	
	Well clearance	0.43 (0.340–0.551)	< 0.001	0.41 (0.315–0.533)	< 0.001

Well clearance, > 0%; less clearance, ≤ 0%

*OR* odds ratio, *CI* confidence interval, *ICU* intensive care unit, *CRRT* continuous renal replacement therapy

^a^Adjusted for age, sex, body weight, septic acute kidney injury, mechanical ventilation, vasopressors, and scores of SOFA, APACHE II, and CCI

## Discussion

The present study assessed the clinical utility of the serum lactate level as a predictive factor of mortality in patients with severe AKI requiring CRRT. Initial hyperlactatemia was associated with the risk of mortality, regardless of disease severity and other baselines. Furthermore, the addition of lactate levels to the severity index score-based models was better in predicting mortality outcomes than the models without lactates. Lactate has been a well-known factor related to poor prognosis in critically ill patients [25, 28, 29]. However, this issue has less been studied in patients with AKI requiring CRRT. Although some previous studies on this group addressed the association between refractory acidosis and mortality which might be mainly attributed to hyperlactatemia [20, 21], we could find few large-scale cohort studies which applicate lactates to predict mortality.

Hyperlactatemia in critical illness reflects the interaction between overproduction and reduced utilization, and its pathogenesis may be multifactorial [2]. Global, or sometimes local, hypoxia results in the overproduction and underutilization of lactate, which compromises mitochondrial oxidation [30]. Nonhypoxemic causes include catecholamine-driven glycolytic flux, stimulation of Na^+^-K^+^-adenylpyrophosphatase activity, and inhibition of pyruvate dehydrogenase, along with decreased lactate metabolism by the liver [31]. The kidneys are also known as major lactate-consuming organs followed by the liver. Lactate is removed during metabolism rather than excretion, which may account for 10% of kidney disposal under severe hyperlactatemia [32]. Hyperlactatemia is a frequent occurrence in patients with severe AKI, therefore supporting its consideration as an early marker of damage according to reduced uptake and metabolism [33, 34].

The interpretation of a single measurement of lactates has certain limitations when compared to serial measurements [5, 26, 35]. A prospective observational study adopted the concept of lactate clearance as a prognostic factor [7], and other studies applied this concept in critical care [27]. Lactate clearance which normally depends on liver gluconeogenesis, could depend more on oxidation in injured, post ischemic or resting tissues during stressful states including sepsis [36]. All extracorporeal removal techniques (e.g., intermittent hemodialysis, charcoal hemoperfusion, and CRRT) may eliminate lactates, but their removal effect seemed to be low in critically ill patients because of the high production rate [37, 38]. It has been suggested that the beneficial effect of CRRT on hyperlactatemia is the improvement in acid–base and metabolic status leading to enhanced lactate metabolism, rather than the direct removal of lactate by ultrafiltration and dialysis [39]. Our result demonstrates that an early trend in serum lactate remains a reliable marker for risk-stratification regardless of the effect of CRRT. One similar study on patients with septic AKI requiring CRRT suggested that lactate clearance and lactate at 24 h after starting CRRT be more useful prognostic markers than initial lactate alone [40]. Compared to the previous study, we used a larger scale of cohort including patients without sepsis and confirmed a single lactate measure at CRRT initiation as a risk-stratification tool.

Although the study is informative, there are certain limitations to be addressed. Because of the retrospective design, unmeasured biases and confounders could have interfered with the present analyses. Continuous fluctuations in biochemical parameters and alterations during practice could be related to mortality but were not considered in the study. The cause of death was not known, which could have affected the results. The causes of hyperlactatemia are generally classified into those associated with impaired tissue hypoxia and those in which systemic hypoperfusion is not apparent but toxin-induced impairment of cellular metabolism might be involve [2]. The present study did not determine the pathophysiology of hyperlactatemia, which could further segment the association between lactate and mortality. The present cohort of patients who underwent CRRT was a particular subgroup, which hindered the applicability of the conclusions to another patient subset.

## Conclusions

Patients with AKI requiring CRRT have a high risk of mortality, and thus, the precise prediction and alarming of critical outcomes are important. The present study identified that hyperlactatemia at CRRT initiation and its reduced clearance were associated with high risk of mortality. Monitoring serum lactate may be needed to precisely predict worse outcomes. The study results provide a basis for future studies that consider lactate levels in patients undergoing CRRT due to AKI.

## Data Availability

The datasets generated during and/or analyzed during the current study are available from the corresponding author on reasonable request.
